# The tetraamine chelator outperforms HYNIC in a new technetium-99m-labelled somatostatin receptor 2 antagonist

**DOI:** 10.1186/s13550-018-0428-y

**Published:** 2018-08-02

**Authors:** Keelara Abiraj, Samer Ursillo, Maria Luisa Tamma, Svetlana N. Rylova, Beatrice Waser, Edwin C. Constable, Melpomeni Fani, Guillaume P. Nicolas, Jean Claude Reubi, Helmut R. Maecke

**Affiliations:** 1 Divisions of Radiopharmaceutical Chemistry and Nuclear Medicine, University Hospital Basel, Petersgraben 4, 4031 Basel, Switzerland; 2 Department of Nuclear Medicine, Medical Centre – University of Freiburg, Faculty of Medicine, University of Freiburg, Hugstetter Strasse 55, 79106 Freiburg, Germany; 3 Division of Cell Biology and Experimental Cancer Research, Institute of Pathology, University of Bern, PO Box 62, Murtenstrasse 31, 3010 Bern, Switzerland; 4 Department of Chemistry, University of Basel, Spitalstrasse 51, 4056 Basel, Switzerland; 5 Roche Pharmaceutical Research and Early Development, Roche Innovation Center Basel, F. Hoffmann-La Roche Ltd, Steinentorberg 8/12, 4051 Basel, Switzerland

**Keywords:** NETs, Somatostatin receptor antagonists, ^99m^Tc, SPECT/CT

## Abstract

**Background:**

Somatostatin receptor targeting radiopeptides are successfully being used to image, stage, and monitor patients with neuroendocrine tumours. They are exclusively agonists that internalise upon binding to the relevant receptor. According to recent reports, antagonists may be preferable to agonists. To date, ^99m^Tc-labelled somatostatin receptor antagonists have attracted little attention. Here, we report on a new somatostatin receptor subtype 2 (sst2) antagonist, SS-01 (p-Cl-Phe-cyclo(D-Cys-Tyr-D-Trp-Lys-Thr-Cys)D-Tyr-NH_2_), with the aim of developing ^99m^Tc-labelled ligands for SPECT/CT imaging. SS-01 was prepared using Fmoc solid-phase synthesis and subsequently coupled to the chelators 1,4,7,10-tetraazacyclododecane-1,4,7,10-tetraacetic acid (DOTA), 6-carboxy-1,4,8,11-tetraazaundecane (N4), and 6-hydrazinonicotinic acid (HYNIC) to form the corresponding peptide-chelator conjugates SS-03, SS-04, and SS-05, respectively. SS-04 and SS-05 were radiolabelled with ^99m^Tc and SS-03 with ^177^Lu. Binding affinity and antagonistic properties were determined using autoradiography and immunofluorescence microscopy. Biodistribution and small animal SPECT/CT studies were performed on mice bearing HEK293-rsst2 xenografts.

**Results:**

The conjugates showed low nanomolar sst2 affinity and antagonistic properties. ^177^Lu-DOTA-SS-01 (^177^Lu-SS-03) and ^99m^Tc-N4-SS-01 (^99m^Tc-SS-04) demonstrated high cell binding and low internalisation, whereas ^99m^Tc-HYNIC/edda-SS-01 (^99m^Tc-SS-05) showed practically no cellular uptake in vitro. The ^99m^Tc-SS-04 demonstrated impressive tumour uptake at early time points, with 47% injected activity per gram tumour (%IA/g) at 1 h post-injection. The tumour uptake persisted after 4 h and was 32.5 %IA/g at 24 h. The uptake in all other organs decreased much more rapidly leading to high tumour-to-normal organ ratios, which was reflected in high-contrast SPECT/CT images.

**Conclusions:**

These data indicate a very promising ^99m^Tc-labelled sst2-targeting antagonist. The results demonstrate high sensitivity of the ^99m^Tc-labelling strategy, which was shown to strongly influence the receptor affinity, contrary to corresponding agonists. ^99m^Tc-SS-04 exhibits excellent pharmacokinetics and imaging properties and appears to be a suitable candidate for SPECT/CT clinical translation.

**Electronic supplementary material:**

The online version of this article (10.1186/s13550-018-0428-y) contains supplementary material, which is available to authorized users.

## Background

Somatostatin receptors are important biomarkers for imaging and targeted radionuclide therapy of human cancers. They belong to the large family of G-protein coupled receptors, which currently account for 30–40% of marketed drugs [1] and are not only overexpressed in neuroendocrine tumours in particular but also in non-neuroendocrine tumours [2]. The receptors make ideal targets for imaging, as they are easily accessible on the plasma membrane of the tumour cell. Their action is mediated through two mechanisms: G-protein activation and β-arrestin function. Among its signalling roles, the latter promotes receptor internalisation—an important mechanism for radiolabelled agonist ligand uptake, accumulation, and retention [3, 4].

In contrast, binding of neutral antagonists does not lead to internalisation but potentially involves many more binding sites than agonist-based approaches. This may lead to the binding of a higher number of radio-vectors and thus to a stronger signal originating from the tumour. Ginj et al. have shown that in both in vitro and in vivo animal models, somatostatin-based radioantagonists may indeed be superior to radioagonists [5]. These findings have recently been duplicated with antagonists conjugated with DOTA and NODAGA and labelled with the positron-emitting radiometals ^68^Ga and ^64^Cu and other 3+ (radio)metals [6–8] and further supported by first-in-human imaging and therapy studies [9–12]. Similarly, preclinical [13–16] and clinical [17] studies of bombesin-based radioantagonists showed that using antagonists may be advantageous over agonists for targeted imaging and therapy of GRP receptor-expressing tumours.

Despite the fact that the first somatostatin-based radioantagonists have been studied in humans and proven to be superior to registered radioagonists, little is known about the influence of bioconjugation, labelling strategies, and other modifications on the in vitro and in vivo pharmacology of radiolabelled antagonists. Wadas et al. compared a potent agonist, TATE ([Tyr^3^,Thr^8^]octreotide) labelled with ^64^Cu using CB-TE2A, a cross-bridged cyclam-14 derivative, with the antagonist (sst2-ANT, p-NO_2_-Phe-cyclo(d-Cys-Tyr-d-Trp-Lys-Thr-Cys)d-Tyr-NH_2_), labelled using the same strategy [18]. They showed that the radioantagonist had lower tumour uptake despite the much higher receptor numbers found in the AR42J tumour model. AR42J cells endogenously express sst2 receptors. The authors also reported a much lower receptor affinity for the antagonist, which might be the reason for the difference rather than the origin of the receptor (natural or transfected), as proposed by the authors.

Even more intriguing is a study by Dude et al. [19], who compared the antagonist ^68^Ga-NODAGA-JR11 with the two agonists ^68^Ga-DOTATOC and ^68^Ga-DOTATATE. Surprisingly, the authors found that ^68^Ga-NODAGA-JR11 has the lowest tumour uptake in their human ZR-75-1 breast tumour model. In addition, the number of receptors using ^68^Ga-DOTATOC was more than twofold higher than ^68^Ga-DOTATATE and significantly higher than the antagonist.

More clinically relevant, however, is the fact that the phenomenon of higher tumour uptake of antagonists was also observed in human tumours. Reubi et al. performed quantitative autoradiography in neuroendocrine and non-neuroendocrine tumour specimens using the ^125^I-DOTA-JR11 antagonist and ^125^I-Tyr^3^octreotide agonist and found up to tenfold higher uptake of the antagonist radioligand compared to the agonist. The authors concluded that all renal cell cancers, most breast cancers, non-Hodgkin lymphomas, and medullary thyroid cancers appear to be novel targets for in vivo targeting with sst2 radioantagonists [20].

These different observations emphasise the need to study the influence of overall structure on pharmacology in vitro and in vivo in different cell and tumour models. In particular, modifications allowing ^99m^Tc-labelling have only been studied in one case [21], although ^99m^Tc-labelled radiopharmaceuticals are still the mainstay of nuclear medicine. We report here the synthesis of a new somatostatin-based antagonist, 4-Cl-Phe-cyclo(D-Cys-Tyr-D-Trp-Lys-Thr-Cys)D-Tyr-NH_2_ (SS-01), which was designed for labelling with ^99m^Tc using two different strategies, namely N4: 6-carboxy-1,4,8,11-tetraazaundecane (SS-04) and HYNIC: 6-hydrazinopyridine-3-carboxylic acid (SS-05), as metal-binding domains. The choice of the chemical structure of the antagonist was based on our previous experiences. We and others observed that the chirality change of amino acids 1 and 2 (aa^1^, aa^2^) and C-terminal amidation compared to octreotide type octapeptides transforms an agonist into an antagonist [22, 23]. In addition, we chose the most easily accessible amino acids leading to antagonistic peptides. To serve as a control, we also modified the peptide with DOTA for labelling with ^177^Lu (^177^Lu-SS-03).

## Methods

The supplier information for reagents, radiolabelling protocols, and log D determination, as well as details about instrumentation, are provided in the Additional file 1.

### Synthesis of chelator-peptide conjugates, radiochemistry

The peptides were assembled on the Rink-Amide methylbenzylhydryl (MBHA) resin employing standard Fmoc strategy. The coupling reactions were performed on a semiautomatic peptide synthesiser (RinkCombichem, Bubendorf, Switzerland) with a threefold excess of Fmoc-amino acids, using DIC/HOBt as activating agents in DMF/NMP for 2 h (see Additional file 1 for details, including labelling protocols and corresponding high-performance liquid chromatography (HPLC) data).

### Binding affinity measurements

Cell membrane pellets were prepared from human sst_1_-expressing Chinese hamster ovary cells (kindly provided by Drs. T. Reisine and G. Singh, University of Pennsylvania, Philadelphia, Pa.); sst_2_-, sst_3_-, and sst_4_-expressing CCL39 cells (kindly provided by Dr. D. Hoyer, Novartis Pharma, Basel, Switzerland); and sst_5_-expressing human embryonic kidney 293 (HEK293) cells (kindly provided by Prof. Stefan Schulz, University of Jena, Germany) and stored at − 80 °C. Quantitative receptor autoradiography was performed on 20-μm-thick membrane pellet sections and quantitated as previously described [22, 24].

### Immunofluorescence microscopy

An immunofluorescence microscopy-based internalisation assay was performed on HEK293-rsst2 cells, as previously described [22]. Briefly, the cells were treated with different antagonist chelator-peptide conjugates and/or [Tyr^3^]octreotide (TOC) (the sst2 agonist) at 37 °C for 30 min. After fixation and permeabilisation, cells were stained with the sst2-specific primary antibody R2-88 (provided by Dr. Agnes Schonbrunn, McGovern Medical School, University of Texas Health Science Center, Houston, Texas, USA) as described previously [25, 26]. The cells were imaged using a Leica DM RB immunofluorescence microscope, and the images were acquired using an Olympus DP10 camera.

### Receptor-binding, internalisation, and dissociation kinetics

The receptor binding, internalisation, and dissociation rates of ^177^Lu-SS-03, ^99m^Tc-SS-04, and ^99m^Tc-SS-05 were studied in HEK293-rsst2 cells seeded in six-well plates, as described previously [6]. Briefly, the radiopeptide (0.25 pmol/well) was added and the cells were incubated at 37 °C. At different time points (0.5, 1, 2, and 4 h), the cellular uptake was stopped by washing twice with ice-cold PBS. The membrane-bound and internalised fractions were collected with ice-cold glycine buffer, pH 2.8 and 1 M NaOH, respectively.

For the dissociation experiments, the plates were placed on ice for 30 min. The radiopeptide (0.25 pmol/well) was added to the cells and allowed to bind for 2 h at 4 °C. The cells were then quickly washed with ice-cold PBS, and fresh pre-warmed (37 °C) medium was added. The cells were incubated at 37 °C for 10, 20, 30, 60, 120, and 240 min. The medium was collected for quantification, and the cells were treated as described previously [6].

The activity in each fraction was measured in a γ-counter (Cobra II). Nonspecific uptake was determined in the presence of 250 pmol/well [Tyr^3^]octreotide. The results were expressed as a percentage of the applied radioactivity.

### Biodistribution studies in HEK293-rsst2-bearing mice

All animal experiments were conducted in compliance with Swiss animal protection laws and associated regulations. The protocol was approved by the Cantonal Veterinary Ethics Committee of the University of Basel (approval #789). Female athymic nude mice (4–6 weeks old) were injected subcutaneously (s.c.) in the right shoulder with 10^7^ HEK293-rsst2 cells in 100 μL sterile PBS. The tumours were allowed to grow for 14–18 days (tumour weight 100–200 mg).

The mice were injected into the tail vein with 100 μL/10 pmol/0.37 MBq of ^177^Lu-SS-03 or ^99m^Tc-SS-04 and were euthanised at 1, 4, and 24 h post-injection (p.i.). Organs of interest and blood were collected, rinsed of excess blood, blotted dry, weighed, and counted in a γ-counter. To determine nonspecific uptake, three animals were pre-injected with 20 nmol of the relevant unlabelled peptide in 0.9% NaCl solution (0.1 mL); after 5 min, the radiopeptide was injected and the percentage of injected activity per gram (%IA/g) was calculated for each tissue. The total counts injected per animal were determined by extrapolation from counts of an aliquot taken from the injected solution as a standard.

### SPECT/CT imaging study

Mice bearing HEK-rsst_2_ tumours were euthanised 4 h after intravenous injection of 15 MBq (150 pmoles) of ^99m^Tc-SS-04 and imaged supine, head first, using a SPECT/CT system dedicated to imaging small animals (NanoSPECT/CT™ Bioscan Inc.). Topogram and helical CT scan of the whole mouse was first acquired using the following parameters: X-ray tube current 177 μA, X-ray tube voltage 45 kVp, 90 s and 180 frames per rotation, pitch 1. The helical SPECT scan was then acquired from head to toe using multi-purpose pinhole collimators (APT1). The energy window width was 20% centred symmetrically over the energy peak of ^99m^Tc at 140 keV. Twenty-four projections (200 s per projection) were used, allowing the acquisition of at least 50 kilocounts/projection.

SPECT images were reconstructed iteratively and filtered using the software package (HiSPECT v1.4.1876, SciVis GmbH, Goettingen, Germany) and the manufacturer’s algorithm (3 subsets, 9 iterations, 35% post-filtering, 128 × 128 matrix, zoom 1, 30 × 20 mm transaxial field of view, resulting in a pixel size of 0.3 mm). CT images were reconstructed using CTReco version r1.146, with a standard filtered back projection algorithm (Exact Cone Beam) and post-filtered (RamLak, 100% frequency cutoff), resulting in a pixel size of 0.2 mm. Co-registered images were visualised in the three orthogonal planes and as maximal intensity projection with InVivoScope v1.43 (Bioscan Inc.).

### Data analysis

Statistical analysis was performed using an unpaired two-tailed *t* test with Prism software (Prism 5.01, September 2007, GraphPad Software Inc.). Differences at the 95% confidence level (*P* < 0.05) were considered significant.

## Results

### Synthesis, radiolabelling, and distribution coefficients (log D)

**Fig. 1:**
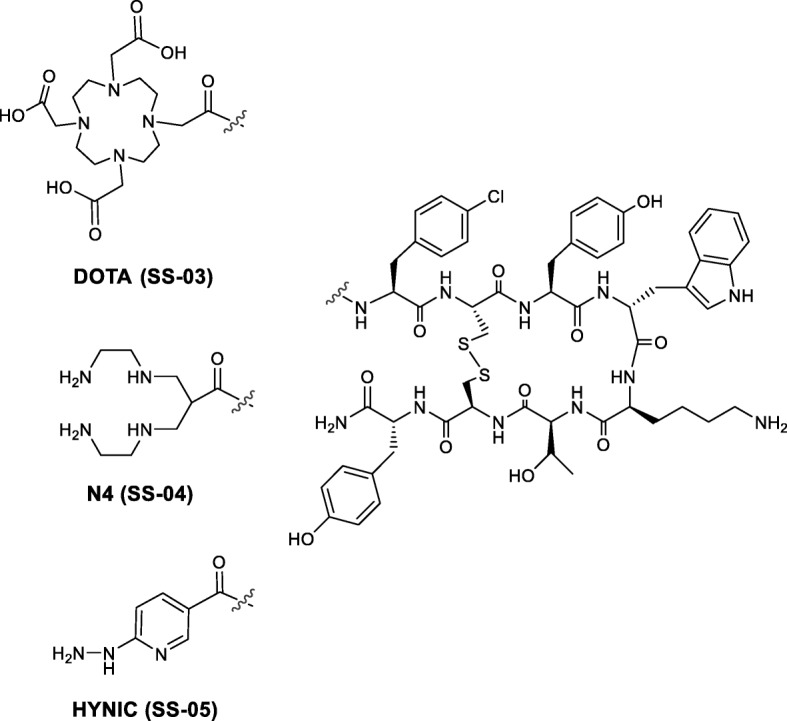
Structures of the somatostatin receptor subtype 2 antagonist conjugated to different chelators

All conjugates (Fig. 1) were synthesised with the maximum yield of 30–40% and purity ≥ 97%. The conjugates were characterised by analytical reversed phase HPLC and ESI-MS (Table 1, Additional file 1).

**Table 1:** Analytical data of the purified chelator-peptide conjugates

Compound	Sequence	Molecular weight (g/mol)	m/z (calc.)	MS (ESI):(m/z)	Purity (%)
SS-03	DOTA-(4-Cl)-Phe-Cyclo(D-Cys-Tyr-D-Trp-Lys-Thr-Cys)-D-Tyr-NH_2_	1531.15	1529.59	767.2 [M+2H]^++^	99.5
				1531.8 [M+H]^+^	
SS-04	N4-(4-Cl)-Phe-Cyclo(D-Cys-Tyr-D-Trp-Lys-Thr-Cys)-D-Tyr-NH_2_	1331.01	1329.56	667.7 [M+2H]^++^	98
				1332 [M+H]^+^	
SS-05	HYNIC-(4-Cl)-Phe-Cyclo(D-Cys-Tyr-D-Trp-Lys-Thr-Cys)-D-Tyr-NH_2_	1279.88	1278.45	642 [M+2H]^++^	97
				1280.8 [M+H]^+^	

SS-03 was labelled with ^177^Lu with labelling yields of > 97% at a maximum specific activity of 50 GBq/μmol. The conjugates SS-04 and SS-05 were labelled with ^99m^Tc at room temperature (30 min) and elevated temperature (95 °C, 10 min), respectively. Tin(II)chloride was used as the reducing agent and citrate as an intermediate supporting Tc(V) ligand for the labelling of SS-04. For SS-05, ^99m^Tc labelling was performed using edda (ethylenediamine, N,N′-diacetic acid) as coligand. The radiolabelling yields of both ^99m^Tc-SS-04 and ^99m^Tc-SS-05 were > 97% at a specific activity of approximately 100 GBq/μmol.

The distribution coefficients (log D) were determined using the shake-flask method (see Additional file 1). All radiopeptides showed high hydrophilicity (^99m^Tc-SS-04, log D = − 2.49 ± 0.34, ^177^Lu-SS-03, log D = − 2.35 ± 0.22 and ^99m^Tc-SS-05, log D = − 2.03 ± 0.27).

### Binding affinity and immunofluorescence microscopy

Table 2 summarises the IC_50_ values of SS-03 and SS-04 for the five somatostatin receptor subtypes (sst1-sst5). Both SS-03 and SS-04 show high selectivity and affinity to sst2. The affinity of SS-03 for the sst2 subtype is threefold higher than SS-04. In comparison with natural somatostatin-28 (SS-28) and the potent sst2 antagonist DOTA-sst2-ANT (DOTA-*p*NO_2_Phe-cyclo[D-Cys-Tyr-D-Trp-Lys-Thr-Cys]-D-Tyr-NH_2_), both SS-03 and SS-04 retained high affinity to sst2.

**Table 2:** Binding affinities (IC_50_, nM) of chelator-peptide conjugates

Compound	IC_50_ (nM)*				
	sst_1_	sst_2_	sst_3_	sst_4_	sst_5_
SS-03	> 1000	1.7 ± 0.06	> 1000	404 ± 92	564 ± 174
SS-04	> 1000	5.3 ± 0.17	720 ± 74	171 ± 35	228 ± 73
DOTA-sst2-ANT [22]	> 1000	1.5 ± 0.4	> 1000	287 ± 27	> 1000
SS-28	5.2 ± 0.3	2.7 ± 0.38	7.7 ± 0.9	5.6 ± 0.4	4.0 ± 0.3

*Values are IC_50_ in nM (mean ± SEM; *n* ≥ 3)

**Fig. 2:**
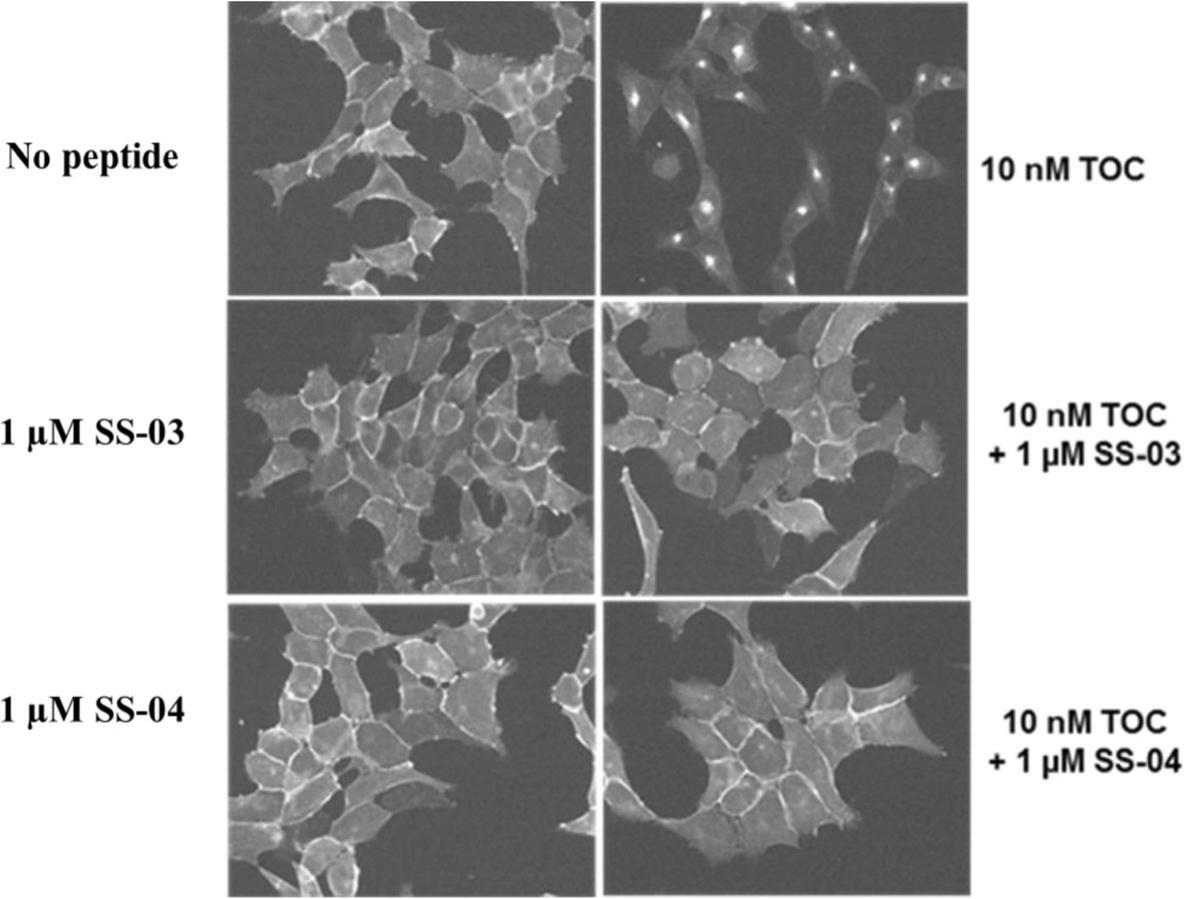
Immunofluorescence microscopy-based internalisation assay on HEK-sst2 cells. Immunofluorescence microscopy-based internalisation assay with HEK-sst2 cells showing the sst2 internalisation induced by [Tyr^3^]octreotide (TOC) is efficiently antagonised by SS-03 and SS-04. Control experiment showing membrane bound sst2-receptor in the absence of peptide. The agonist, TOC, triggered massive sst2-receptor internalisation at a concentration of 10 nM. The antagonists SS-03 and SS-04 failed to induce sst2 internalisation even at a concentration of 1 μM. Further, both SS-03 and SS-04 at a concentration of 1 μM efficiently blocked the agonist (TOC, 10 nM)-mediated sst2-receptor internalisation

Immunofluorescence-based internalisation was performed using HEK-sst2 cells to demonstrate the antagonistic property of the conjugates. Figure 2 illustrates that 10 nM of the agonist [Tyr^3^]octreotide (TOC) triggers massive receptor internalisation, whereas SS-03 or SS-04 at the much higher concentration of 1000 nM does not stimulate receptor internalisation. However, at a concentration of 1 μM together with 10 nM of TOC, the conjugates were able to prevent the agonist-induced receptor internalisation.

### Receptor-binding, internalisation, and dissociation kinetics

**Fig. 3:**
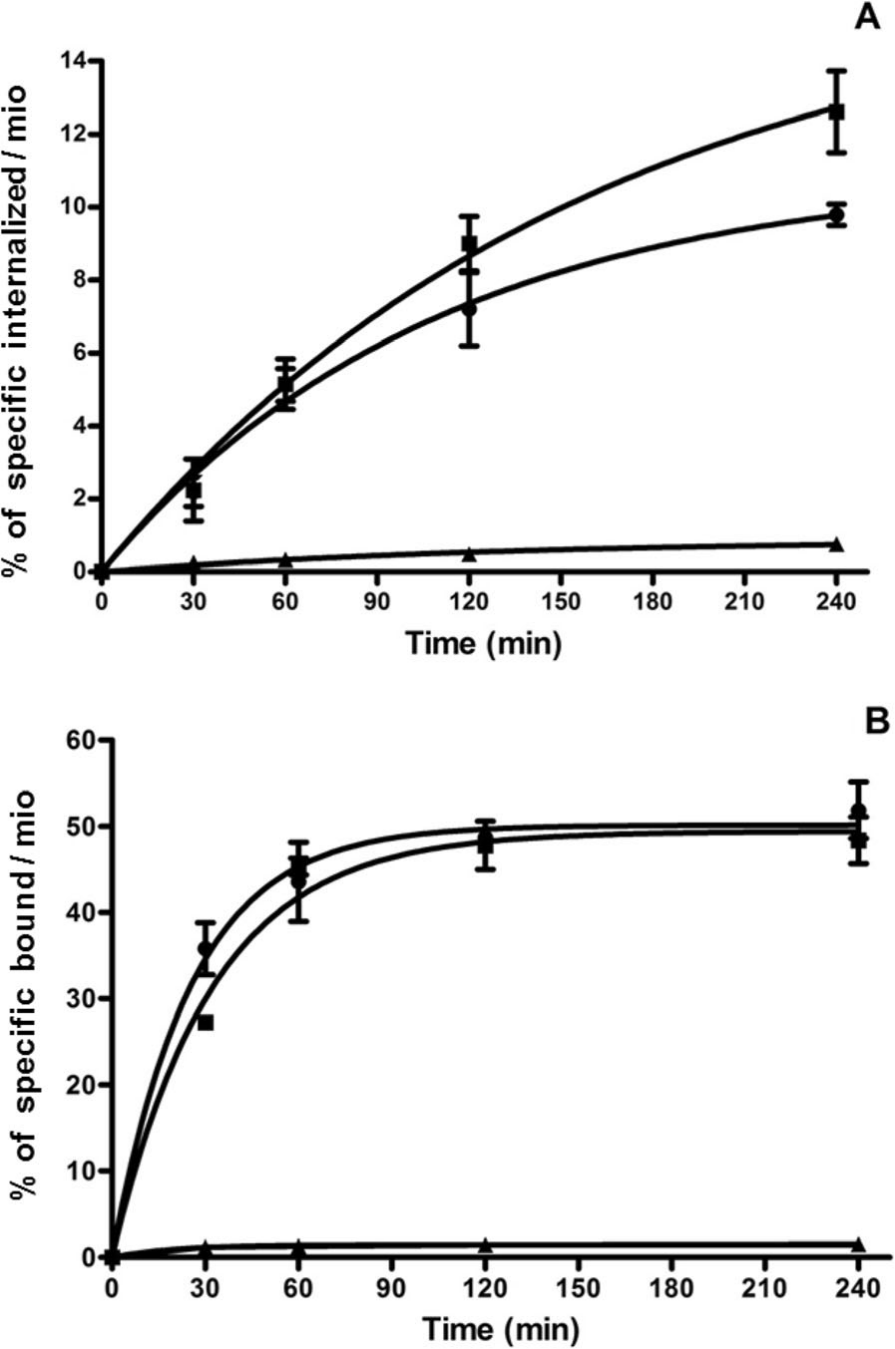
In vitro cellular uptake profile of the radioligands on HEK293-rsst2 cells. Cellular uptake profile of ^177^Lu-SS-03 (circle), ^99m^Tc-SS-04 (square), and ^99m^Tc-SS-05 (triangle) as measured with HEK293-rsst2 cells. **a** The amount of specifically internalised and **b** radiopeptides specifically bound to the membrane. Values and standard deviations are the result of two independent experiments with triplicates in each experiment

Figure 3 shows the cellular uptake profiles of the radioligands as measured with HEK-rsst2 cells. ^177^Lu-SS-03 and ^99m^Tc-SS-04 showed high uptake and blocking studies demonstrated that the uptake was receptor-mediated. Within 2 h of incubation, the specifically bound fraction levelled off at 48%, demonstrating rapid binding of ^177^Lu-SS-03 and ^99m^Tc-SS-04 to the receptors. The internalised fraction was about 8–10% and increased very slowly. Surprisingly, ^99m^Tc-SS-05 showed very low cellular uptake of < 1% even after 4 h of incubation.

**Fig. 4:**
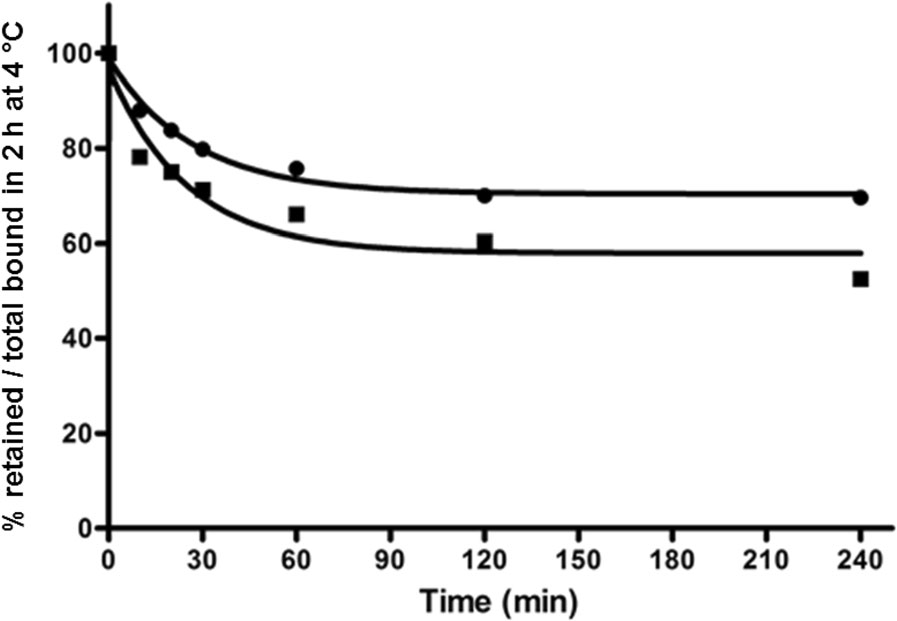
Dissociation kinetics of the cell surface bound radioligands on HEK293-rsst2 cells. Dissociation kinetics of membrane bound ^177^Lu-SS-03 (circle) and ^99m^Tc-SS-04 (square) as measured with HEK293-rsst2 cells. The profile illustrates the varying degree of dissociation kinetics exhibited by ^177^Lu-SS-03 (koff = 0.037 min^-1^ and t1/2 = 18.66 min) and ^99m^Tc-SS-04 (koff = 0.040 min^-1^ and t1/2 = 17.24 min). Values and standard deviations are the result of two independent experiments with triplicates in each experiment. The rate constants were determined by fitting to a pseudofirst order reaction using GraphPad Prism

The dissociation kinetics of ^177^Lu-SS-03 and ^99m^Tc-SS-04 were studied by a temperature shift experiment and showed a plateau at 2 h, indicating that a steady state had been achieved (Fig. 4). Both conjugates bind strongly to the receptor, displaying only 30 and 48% of dissociation, respectively, after 4 h at 37 °C.

### Biodistribution studies and SPECT imaging

Table 3 summarises the biodistribution of ^177^Lu-SS-03 and ^99m^Tc-SS-04 evaluated at 1, 4, and 24 h after injection in HEK-rsst2 xenografts. At 1 h, ^99m^Tc-SS-04 showed high uptake in the liver (7.18 ± 0.40 %IA/g) and kidneys (49.82 ± 4.31 %IA/g) and higher blood pool activity (1.85 ± 0.41 %IA/g) than ^177^Lu-SS-03 (liver 3.00 ± 0.66 %IA/g, kidneys 15.15 ± 1.86 %IA/g, and blood 0.57 ± 0.04 %IA/g). ^99m^Tc-SS-04 exhibits twofold higher tumour uptake compared to ^177^Lu-SS-03. Uptake in other sst2 receptor-positive organs such as the stomach and pancreas is lower for ^99m^Tc-SS-04 (11.1 ± 2.9 and 15.6 ± 0.8 %IA/g, respectively) than ^177^Lu-SS-03 (48.7 ± 13.1 and 72.6 ± 13.8 %IA/g, respectively). At 4 h, the tumour uptake of ^177^Lu-SS-03 increased to 31.68 ± 4.00 %IA/g, whereas the tumour uptake of ^99m^Tc-SS-04 remained constant. Tumour uptake of ^177^Lu-SS-03 and ^99m^Tc-SS-04 was significantly reduced by pre-injection of 20 nmol of unlabelled peptide. At 24 h, both conjugates showed clearance from the blood pool (< 0.1 %IA/g) and significant wash-out from the liver (^177^Lu-SS-03: 0.47 ± 0.07 %IA/g and ^99m^Tc-SS-04: 1.87 ± 0.33 %IA/g) and kidneys (^177^Lu-SS-03: 7.3 ± 1.6 %IA/g and ^99m^Tc-SS-04: 6.3 ± 1.8 %IA/g). ^99m^Tc-SS-04 showed higher tumour uptake at all time points including 24 h p.i. (32.5 ± 0.78 %IA/g) compared to ^177^Lu-SS-03 (26.32 ± 4.42 %IA/g). The tumour-to-normal tissue ratios of ^177^Lu-SS-03 and ^99m^Tc-SS-04 at each time point are illustrated in Table 4. At 1 h, ^177^Lu-SS-03 shows higher tumour-to-normal tissue ratios compared to ^99m^Tc-SS-04. At 4 h, both radiopeptides showed similar ratios except ^177^Lu-SS-03 had a twofold higher tumour-to-liver ratio. However, at 24 h, ^99m^Tc-SS-04 exhibited excellent biodistribution with a 4.5-fold higher tumour-to-blood ratio, approximately threefold higher tumour-to-kidney ratio, and approximately twofold higher tumour-to-liver ratio compared to ^177^Lu-SS-03. All relevant tumour-to-normal organ ratios increased with time and were > 4 at 24 h.

**Table 3:** Biodistribution results of ^177^Lu-SS-03 and ^99m^Tc-SS-04 in nude mice bearing HEK293-rsst2 tumour xenografts. Data expressed as %IA/g (percentage of injected activity per gram) and presented as mean ± SD (*n* = 3–5)

Organs	1 h	4 h	4 h blocking*	24 h
^177^Lu-SS-03				
Blood	0.57 ± 0.04	0.21 ± 0.04	0.07 ± 0.02	0.07 ± 0.04
Heart	0.69 ± 0.12	0.38 ± 0.07	0.11 ± 0.01	0.11 ± 0.04
Liver	3.00 ± 0.66	1.75 ± 0.22	1.04 ± 0.09	0.47 ± 0.07
Spleen	1.51 ± 0.19	1.42 ± 0.77	0.28 ± 0.04	0.33 ± 0.06
Lung	13.24 ± 4.49	6.71 ± 1.37	0.59 ± 0.11	0.74 ± 0.22
Kidney	15.15 ± 1.86	17.04 ± 1.81	20.24 ± 4.30	7.35 ± 1.66
Stomach	48.74 ± 13.11	30.21 ± 4.25	0.37 ± 0.06	6.02 ± 1.40
Intestine	3.13 ± 1.16	2.28 ± 0.31	0.24 ± 0.03	0.27 ± 0.06
Adrenal	3.70 ± 0.63	2.90 ± 0.46	0.06 ± 0.03	1.16 ± 0.46
Pancreas	72.61 ± 13.77	54.49 ± 7.28	0.24 ± 0.07	3.03 ± 0.62
Muscle	0.22 ± 0.06	0.12 ± 0.02	0.06 ± 0.00	0.07 ± 0.02
Bone	4.11 ± 0.53	2.77 ± 1.06	0.12 ± 0.03	1.25 ± 0.16
Tumour	23.64 ± 1.28	31.68 ± 4.00	11.15 ± 1.93	26.32 ± 4.42
^99m^Tc-SS-04				
Blood	1.85 ± 0.41	0.22 ± 0.03	0.22 ± 0.01	0.03 ± 0.01
Heart	1.42 ± 0.30	0.25 ± 0.06	0.23 ± 0.02	0.09 ± 0.01
Liver	7.18 ± 0.40	5.13 ± 0.51	4.07 ± 0.65	1.87 ± 0.33
Spleen	2.09 ± 0.34	0.85 ± 0.08	0.63 ± 0.02	0.41 ± 0.11
Lung	15.93 ± 2.69	2.03 ± 0.14	2.00 ± 0.37	0.54 ± 0.10
Kidney	49.82 ± 4.31	25.56 ± 1.01	18.48 ± 3.66	6.31 ± 1.82
Stomach	11.13 ± 2.98	1.99 ± 0.39	0.56 ± 0.03	0.62 ± 0.14
Intestine	2.12 ± 0.57	0.57 ± 0.06	0.42 ± 0.03	0.16 ± 0.02
Adrenal	3.17 ± 0.69	0.99 ± 0.20	0.37 ± 0.10	0.50 ± 0.13
Pancreas	15.58 ± 0.82	1.16 ± 0.86	0.33 ± 0.06	0.31 ± 0.11
Muscle	0.64 ± 0.13	0.12 ± 0.02	0.12 ± 0.02	0.05 ± 0.03
Bone	3.15 ± 0.72	0.92 ± 0.15	0.40 ± 0.01	0.44 ± 0.23
Tumour	47.14 ± 7.23	47.24 ± 7.96	14.17 ± 1.69	32.51 ± 0.78

^*^Pre-injection with 20 nmol of unlabelled peptide (SS-03 or SS-04)

**Table 4:** Tumour-to-normal tissue ratios of ^177^Lu-SS-03 and ^99m^Tc-SS-04 after 1, 4, and 24 h of administration in nude mice bearing HEK293-rsst2 tumour xenografts

	^177^Lu-SS-03			^99m^Tc-SS-04		
	1 h	4 h	24 h	1 h	4 h	24 h
Tumour/blood	41.3	150.5	360.9	25.5	218.1	981.3
Tumour/liver	7.9	18.1	55.8	6.6	9.2	17.4
Tumour/kidney	1.56	1.86	3.58	0.95	1.85	5.16
Tumour/muscles	109.5	259.3	372.1	73.6	382.1	616.7
Tumour/bone	5.7	11.5	21.1	14.9	51.3	74.1

**Fig. 5:**
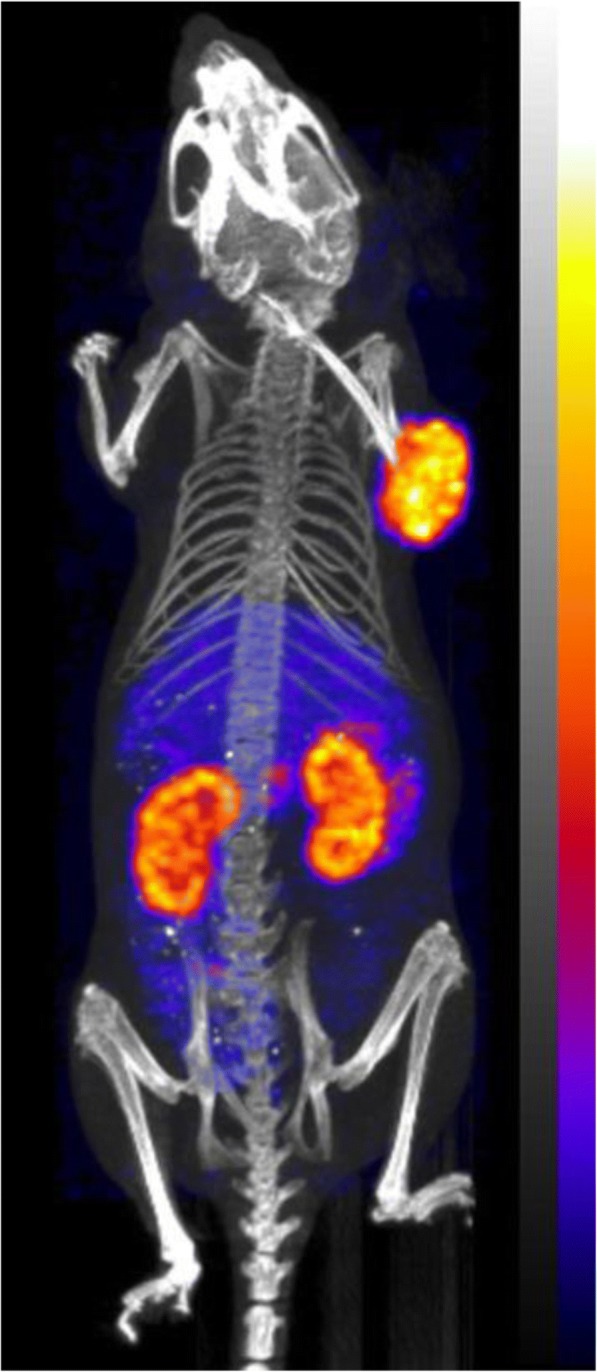
SPECT/CT imaging of ^99m^Tc-SS-04 on HEK-rsst2-xenograft-bearing mouse. Maximum intensity projection (MIP) of the nanoSPECT/CT image acquired 4 h after injection of 15 MBq (150 pmol peptide) ^99m^Tc-SS-04 in a HEK-rsst2-xenograft-bearing mouse. The colour bar corresponds to 0–90%IA/g

The SPECT/CT images (Fig. 5) were acquired at 4 h after intravenous injection of ^99m^Tc-SS-04. The highest uptake was visible in the tumour and the kidneys. To a lesser extent, uptake was also visible in the abdomen due to tracer accumulation in the sst2-expressing organs.

## Discussion

Radiolabelled peptides targeting G-protein-coupled receptors have been an important focus in the radiopharmaceutical and nuclear oncology fields over the last 20 years [27–29]. The clinically employed somatostatin receptor-targeting peptides are agonists, which exhibit nanomolar binding affinities and fast receptor-mediated internalisation in vitro and in vivo via endocytosis. More recently, sst2 antagonists were studied in animal models [6–8, 18] and in humans [9–12]. Surprisingly, the antagonists showed high and long-lasting tumour uptake in most cases. However, antagonist superiority cannot be generalised, as shown by Wadas et al. [18] and Dude et al. [19].

To date, many peptides labelled with gamma-, positron-, and beta-emitters (^111^In, ^64^Cu, ^68^Ga, and ^177^Lu) have been described, but only one ^99m^Tc-labelled antagonist has been reported to date [21].

We selected two of the more popular and successful chelators for ^99m^Tc: HYNIC with the co-ligand edda (ethylenediamine-N,N′-diacetic acid) and the bifunctional tetraamine N4. The HYNIC core has been widely used for the labelling of octreotide-based somatostatin receptor-targeting peptides, and the [HYNIC, Tyr^3^]octreotide/edda kit (HYNIC-TOC) is registered in some European countries. The [O=Tc=O]^+^ core is formed with linear and macrocyclic tetraamines such as 1,4,8,11-tetraazaundecane and cyclam-14. This core exhibits high kinetic stability, can be labelled at room temperature, is highly hydrophilic, and with different functions in 6-position, such as carboxylic acid can be easily coupled to the N-termini of biomolecules [30]. We have conjugated these two chelators to a new antagonist, p-Cl-Phe-cyclo(D-Cys-Tyr-D-Trp-Lys-Thr-Cys)D-TyrNH_2_. As mentioned earlier, we chose the most easily accessible amino acids leading to antagonistic peptides. Our new antagonist is based on a modification of the first radiolabelled sst2 antagonists (DOTA-BASS) [5, 9, 31], where the p-NO_2_-Phe has been replaced by p-Cl-Phe. We also conjugated DOTA to this antagonist and labelled the conjugate with ^177^Lu for comparative in vitro and in vivo studies.

The antagonistic properties of SS-03 and SS-04 were investigated using immunofluorescence microscopy; the two compounds inhibit receptor internalisation triggered by the potent agonist [Tyr^3^]octreotide but do not trigger internalisation on their own. Somewhat surprisingly, but as previously reported [6, 14, 17], we observed low but significant cell internalisation of ^177^Lu-SS-03 and ^99m^Tc-SS-04 (around 10% of the added radiopeptide activity per one million cells at 4 h). This uptake can be blocked by excess unlabelled peptide. The internalisation rate is significantly lower than that of somatostatin-based radiolabelled agonists [14, 22, 32]. The reason for the difference between the two assays is unclear.

A very important outcome of this work is that [^99m^Tc-HYNIC/edda]-SS-01 shows practically no cell binding or internalisation in our HEK-rsst2 cell assay. This is contrary to HYNIC conjugated to somatostatin-based agonistic octapeptides such as [Tyr^3^]octreotide (TOC) and [Tyr^3^,Thr^8^]octreotide (TATE). These conjugates showed impressive preclinical [3] and clinical pharmacokinetics [33–35]. The influence of chelator and even radiometal on the pharmacologic properties was also reported for DOTA- and NODAGA-conjugated sst2 antagonists [6, 7].

In contrast, the N4-conjugated and ^99m^Tc-labelled radiopeptide showed superior properties, with very high cell uptake in vitro and about the highest tumour uptake at 1 and 4 h of any somatostatin-based radiopeptide studied to date in this xenograft model [6, 7]. The higher tumour uptake of ^99m^Tc-SS-04, compared with ^177^Lu-SS-03, may be attributed to the somewhat slower blood clearance of ^99m^Tc-SS-04, even though at 4 h p.i, the blood values are at the same level for both radiopeptides. In addition, it shows very good tumour retention, no change between 1 and 4 h, and only about 30% release between 1 and 24 h p.i. The target-to-relevant organ ratios are > 5 at 24 h, reflected in excellent SPECT/CT images. For both radiotracers, the kidney uptake is somewhat high but the tumour-to-kidney ratios are still within a reasonable range when compared to potent radioagonists. We are currently studying somewhat different peptide motifs coupled to tetraamine chelators for ^99m^Tc-labelling to improve the overall pharmacokinetics. The hypothesised improvement is based on the properties of ^177^Lu-SS-03, which exhibits pharmacokinetics somewhat inferior to the previously published ^111^In-DOTA and ^177^Lu-DOTA conjugated antagonists [31]. As previously mentioned, Radford et al. studied a somewhat different sst2 antagonist peptide labelled using the ^99m^Tc tricarbonyl strategy and an NSN-type chelator [21]. The Re-complexed congener showed reasonably good receptor affinity indicating that this strategy, other than the HYNIC strategy, retains binding affinity [21]. The problem with the radioligand is its high abdominal uptake and low tumour-to-normal organ ratios, which is below 1 for some relevant tissues. As the authors pointed out, this is due to the high lipophilic character of the radioligand. The new ^99m^Tc-based sst2 antagonist ^99m^Tc-SS-04 is hydrophilic, as indicated by its log D value. Consequently, it has limited accumulation in the abdomen and rather high renal excretion. The biodistribution profile of ^99m^Tc-SS-04, together with its very high tumour uptake, favours this tracer for clinical translation.

## Conclusions

Sst2 antagonists were labelled with ^99m^Tc using two common chelators. Surprisingly, the widely used ^99m^Tc ligand HYNIC along with the co-ligand edda resulted in almost complete loss of sst2 binding affinity, whereas ^99m^Tc-SS-04 demonstrated impressive tumour uptake and high tumour-to-normal organ ratios. Therefore, ^99m^Tc-SS-04 appears to be an excellent candidate for SPECT imaging of sst2-positive tumours and is a promising option for further modification of the peptide motif. The pharmacokinetics of ^99m^Tc-SS-04 demonstrate once again that the ^99m^TcO_2_(N4) core offers favourable pharmacokinetic features for small peptides and may potentially be the ^99m^Tc-labelling strategy of choice.

## Additional file

Additional file 1: Materials and methods. (PDF 484 kb)

## Abbreviations

%IA/g: Injected activity per gram
DIC: *N,N′*-Diisopropylcarbodiimide
DMF: N,N-Dimethylformamide
edda: Ethylenediamine,N,N′-diacetic acid
Fmoc: Fluorenylmethyloxycarbonyl
GPCR: G-protein coupled receptors
GRP: Gastrin-releasing peptide
HEK: Human embryonic kidney
HOBt: Hydroxybenzotriazole
HPLC: High-performance liquid chromatography
HYNIC: 6-hydrazinonicotinic acid
IC_50_: Half maximal inhibitory concentration
NETs: Neuroendocrine tumours
NMP: N-methylpyrrolidinone
p.i.: Post-injection
PBS: Phosphate-buffered saline
s.c.: Subcutaneously
SPECT/CT: Single-photon emission computed tomography/computed tomography
sst: Somatostatin receptor
sst2: Somatostatin receptor subtype 2
TATE: [Tyr^3^,Thr^8^]octreotide
TOC: [Tyr^3^]octreotide
CB-TE2A: 4,11-bis(carboxymethyl)-1,4,8,11-tetraazabicyclo[6.6.2]hexadecane
NODAGA: 1,4,7-triazacyclononane,1-glutaric acid-4,7-acetic acid
DOTA: 1,4,7,10-tetraazacyclododecane-1,4,7,10-tetraacetic acid
N4: 6-carboxy-1,4,8,11-tetraazaundecane
