# Supplementary material for: The tetraamine chelator outperforms HYNIC in a new technetium-99m-labelled somatostatin receptor 2 antagonist
Source: EJNMMI Res. 2018 Aug 2;8:75. doi: 10.1186/s13550-018-0428-y (PMC6070450; doi:10.1186/s13550-018-0428-y)
Supplement: Supplementary file 1 — Materials and methods. (PDF 484 kb) [file 13550_2018_428_MOESM1_ESM.pdf]

The tetraamine chelator outperforms HYNIC in a new technetium-99m-labelled somatostatin receptor 2 antagonist

Additional file 1

Keelara Abiraj[1,§,#], Samer Ursillo[1,#], Maria Luisa Tamma[1], Svetlana N. Rylova[2], Beatrice Waser[3], Edwin C. Constable[4], Melpomeni Fani[1], Guillaume P. Nicolas[1], Jean Claude Reubi[3], Helmut R. Maecke[1,2¶]

[1]Divisions of Radiopharmaceutical Chemistry and Nuclear Medicine, University Hospital Basel, Petersgraben 4, 4031, Basel, Switzerland

[2]Department of Nuclear Medicine, Medical Centre – University of Freiburg, Faculty of Medicine, University of Freiburg, Hugstetter Strasse 55, 79106 Freiburg, Germany

[3]Division of Cell Biology and Experimental Cancer Research, Institute of Pathology, University of Bern, PO Box 62, Murtenstrasse 31, 3010, Bern, Switzerland

[4]Department of Chemistry, University of Basel, Spitalstrasse 51, 4056, Basel, Switzerland

[§]Present Address: Roche Pharmaceutical Research and Early Development, Roche Innovation Center Basel, F. Hoffmann-La Roche Ltd, Steinentorberg 8/12, 4051, Basel, Switzerland

[#]Authors contributed equally to this work

Corresponding author: Prof. Helmut R. Maecke

Dr. Keelara Abiraj (abiraj.keelara@roche.com)
Samer Ursillo (samer.ursillo@gmail.com)
Dr. Maria Luisa Tamma (marialuisa.tamma@virgilio.it)
Dr. Svetlana N. Rylova (rylova@hotmail.com)
Beatrice Waser (waserpatho@rubigen.ch)
Prof. Edwin C. Constable (edwin.constable@unibas.ch)
Prof. Melpomeni Fani (melpomeni.fani@usb.ch)
Dr. Guillaume P. Nicolas (guillaume.nicolas@usb.ch)
Prof. Jean Claude Reubi (reubi@pathology.unibe.ch)
Prof. Helmut R. Maecke (helmut.maecke@uniklinik-freiburg.de)

Additional File 1: Materials and methods

Reagents and Instrumentation

All reagents were obtained from commercial sources and used without further purification. The amino acids, were obtained from Bachem AG (Bubendorf, Switzerland) or Novabiochem (Laeufelfingen, Switzerland). DOTA(^{t}Bu)$_3$ (1,4,7,10-tetraazacyclododecane-1,4,7-tris(acetic acid-*t*-butyl ester)-10-acetic acid) was obtained from CheMatech (Dijon, France). Boc-HYNIC (6-Boc-hydrazinopyridine-3-carboxylic acid) was synthesized according to Abrams et al. (*1*) Boc protected tetramine-chelator, (Boc)$_4$-N4 (*N,N',N'',N'''*-tetrakis(*tert*-butyloxycarbonyl)-6-(carboxy)-1,4,8,11-tetraazaundecane), was synthesized according to the procedure described previously (*2*). $^{177}LuCl_3$ was obtained from IDB (Baarle-Nassau, The Netherlands). ^{99m}Tc was eluted as Na[^{99m}Tc]TcO$_4$ from a $^{99}Mo/^{99m}Tc$ generator (Mallinckrodt, Petten, the Netherlands). Electrospray ionisation (ESI) mass spectroscopy (MS) was carried out with a Finnigan SSQ7000 (Bremen, Germany). Analytical high-performance liquid chromatography (HPLC) was performed on a Hewlett Packard 1050 HPLC system with a multiwavelength detector and a flow-through Berthold LB 506 Cl γ-detector using a Macherey-Nagel Nucleosil 120 C18 column. Preparative HPLC was performed on a Metrohm HPLC system LC-CaDI 22–14 with a Macherey-Nagel VP 250/21 Nucleosil 100–5 C18 column. Both analytical and preparative columns were eluted with a gradient system of mixtures of aqueous 0.1% TFA (solvent A) and acetonitrile (solvent B). Quantitative γ-counting was performed on a COBRA 5003 γ-system well counter from Packard Instruments.

Synthesis of Chelator-Peptide Conjugates

The peptides were assembled on Rink-Amide MBHA resin employing standard Fmoc strategy.(*3*). The coupling reactions were achieved with 3-fold excess of Fmoc-amino acids, using DIC/HOBt as activating agents in DMF/NMP for 2 h, on a semiautomatic peptide synthesizer (RinkCombichem, Bubendorf, Switzerland). Fmoc removal was achieved with 20% piperidine in DMF in three successive 10 min treatments. Cyclization was performed afterwards using thallium(III) trifluoroacetate ($Tl(CF_3CO_2)_3$).

The cyclized resin assembled with desired amino acids was divided into three parts after the removal of the final Fmoc group. The chosen prochelators (DOTA(^{t}Bu)$_3$, (Boc)$_4$-N4 or Boc-HYNIC) were coupled to the *N*-terminal of the peptide on resin using HATU (3.3 equivalents) and DIPEA (7 equivalents) for 4 h. In case of HYNIC, 6 equivalents of HATU and 12

equivalents of DIPEA were used. The cleavage and deprotection of the peptide-chelator conjugates was accomplished by incubating for 1.5h in TFA:thioanisole:triisopropylsilane:H_2O 95:2:2:1. The resin was then filtered and washed with the above mixture, after which the filtrate was evaporated and triturated with diethyl ether to give the crude product. This was purified by semi-preparative HPLC to obtain the corresponding chelator-peptide conjugate with purity ≥97%.

Radiolabeling of Chelator-Peptide Conjugates

^{177}Lu-SS-03: 10 µg of SS-03 in 300 µL sodium acetate buffer (0.4 mol/L, pH 5.0) were incubated with $^{177}LuCl_3$ (74-92 MBq) for 30 min at 95°C.

^{99m}Tc-SS-04: Na[^{99m}Tc]TcO_4 (555-740 MBq, 700 µL) was added to a mixture of 0.5 M phosphate buffer pH 11.5 (50 µL) and 0.1 M sodium citrate (5 µL) followed by SS-04 solution (30 µg, 25 µL) and freshly prepared $SnCl_2$ solution in ethanol (30 µg, 25 µL). The reaction mixture was incubated at room temperature for 30 min, according to the published protocol (*4*) .

^{99m}Tc-SS-05: **30** µg SS05 was added to 1 mL aqueous edda/tricine solution (5 mg edda, 15 mg tricine). 500 µL Na$^{99m}TcO_4$ eluate (555-740 MBq) was added along with 40 µg (20 µL) $SnCl_2$/HCl (0.1 M) solution. The pH was adjusted to 7 by adding 1M NaOH. All manipulations were performed under an Ar tend. The reaction mixture was incubated for 10 min at 95°C. All radiolabeled peptides were analyzed with analytical HPLC.

Determination of distribution coefficients (logD)

Each radiopeptide was added to a presaturated 1/1mixture of PBS/n-octanol, at a concentration of 100 nmol/L. The mixtures were vigorously shaken for 10 min and then centrifuged. The activity concentrations of equal volume samples of both phases were measured in a γ-counter and the partition coefficient (LogD) was calculated.

References

1 Abrams MJ, Juweid M, tenKate CI, et al. Technetium-99m-human polyclonal IgG radiolabeled via the hydrazino nicotinamide derivative for imaging focal sites of infection in rats. *J Nucl Med.* 1990;31:2022-2028.

2. Abiraj K, Mansi R, Tamma ML, et al. Tetraamine-derived bifunctional chelators for technetium-99m labelling: synthesis, bioconjugation and evaluation as targeted SPECT imaging probes for GRP-receptor-positive tumours. *Chem-Eur J.* 2010;16:2115-2124.
3. Atherton E, Sheppard R. Fluorenylmethoxycarbonyl-polyamide solid phase peptide synthesis. General principles and development. *Solid phase peptide synthesis. A practical approach*. Oxford: Oxford Information Press; 1989:25-38.
4. Nock B, Nikolopoulou A, Chiotellis E, et al. [^{99m}Tc]Demobesin 1, a novel potent bombesin analogue for GRP receptor-targeted tumour imaging. *Eur J Nucl Med Mol Imaging.* 2003;30:247-258.

Supplementary figures

A

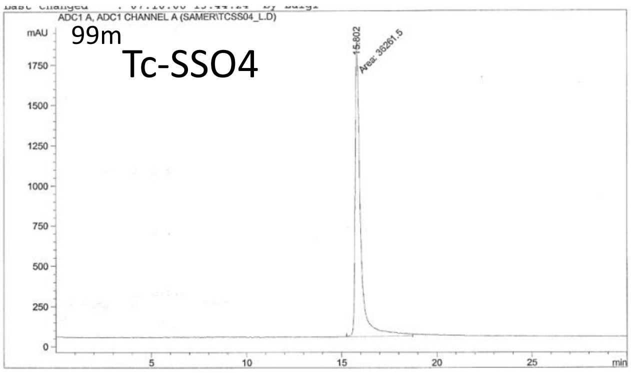

B

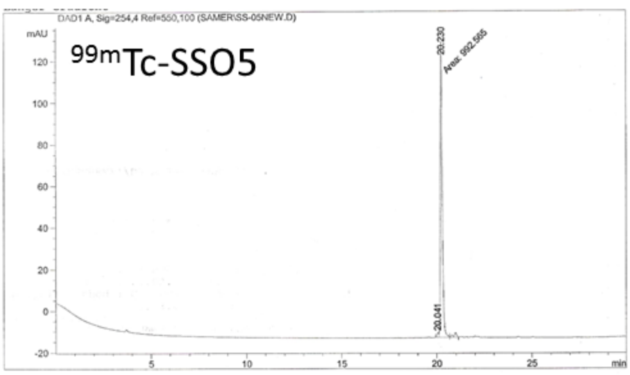

C

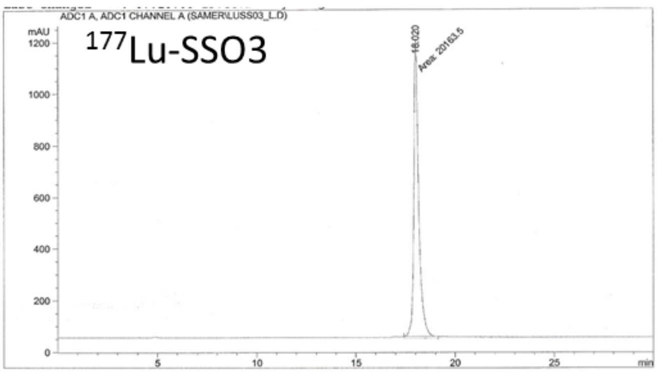

Supplementary Figure 1.
Radio HPLC chromatograms showing the radiochemical purity of ^{99m}Tc-SS-04 (A), ^{99m}Tc-SS-05 (B), ^{177}Lu-SS-03 (C).
